# *Luzonichthys seaver*, a new species of Anthiinae (Perciformes, Serranidae) from Pohnpei, Micronesia

**DOI:** 10.3897/BDJ.3.e4902

**Published:** 2015-04-27

**Authors:** Joshua M. Copus, Cassie A Ka'apu-Lyons, Richard L. Pyle

**Affiliations:** ‡ Hawaii Institute of Marine Biology, Kaneohe, United States of America; § University of Hawaii Manoa, Honolulu, United States of America; | Bishop Museum, Honolulu, Honolulu, United States of America

**Keywords:** Serranidae, Luzonichthys, new species, Mesophotic Coral Ecosystems, MCE, Pohnpei, Micronesia

## Abstract

*Luzonichthys seaver*, n. sp., is described from two specimens, 42-46 mm standard length (SL) collected from Pohnpei, Micronesia. Collections were made by divers on mixed-gas closed-circuit rebreathers using hand nets at depths of 90-100 m. *Luzonichthys seaver* is distinct from all other species of the genus in the characters of lateral line scales, gill rakers, pelvic fin length, caudal concavity and coloration. Of the six species of *Luzonichthys*, it appears to be morphologically most similar to *L. earlei* and *L. whitleyi*.

## Introduction

The genus *Luzonichthys* Herre 1936 consists of six species of small, slender serranids within the subfamily Anthiinae, distributed throughout the tropical Indo-Pacific. The genus is distinguished from other anthiine genera in general body size and shape, and in possessing two fully separated dorsal fins, two opercular spines, and 11+15 vertebrae (Randall 1981, Randall and McCosker 1992). The currently recognized species include *L. earlei* Randall 1981, *L. microlepis* (Smith 1955), *L. taeniatus* Randall and McCosker 1992, *L. waitei* (Fowler 1931), *L. whitleyi* (Smith 1955), and *L. williamsi* Randall and McCosker 1992. Fowler 1931 originally established *L. waitei* within the genus *Mirolabrichthys* Herre 1927. Herre (1936) established the genus *Luzonichthys* with *waitei* as the type species, but classified it (as did Fowler 1931) within the family Pomadasyidae. Whitley and Colefax 1938 described the new genus and species *Naurua waitei*, and Smith 1955, unaware of Herre's genus *Luzonichthys*, described two additional species (*microlepis* and *addisi*) within *Naurua*, also placing *M. waitei* Fowler 1931 in this genus, thereby establishing *N. waitei* Whitley and Colefax 1938 as a secondary homonym of *M. waitei* Fowler 1931. Smith (1955) proposed the new name *N. whitleyi* as a replacement for Whitley and Colefax's species. Smith (1956) later reassigned the two species he described to *Luzonichthys* and suggested the two other species of *Naurua* may also belong to that genus. Fourmanoir (1977) proposed the species *L. robustus* from seven specimens taken from Mare, Loyalty Islands and one specimen from Kwajalein, Marshall Islands. Randall (1981) described the species *L. earlei* from specimens taken off Oahu, Hawaii and suggested that *L. addisi* and *L robustus* were junior synonyms of *L. waitei*, noting the type series of *robustus* included 4 different species of *Luzonichthys*, the holotype being *L. waitei*. Finally, in a revision of the genus *Luzonichthys*, Randall and McCosker (1992) described two additional new species of the genus, *L. taeniatus* and *L. williamsi*. With the description of the new species, *L. seaver* herein, the number of recognized species within *Luzonichthys* is raised to seven.

## Materials and methods

Type specimens of the new species, *Luzonichthys seaver*, were collected at Pohnpei, Micronesia by hand net from depths of 90-100 m and deposited in the Bernice P. Bishop Museum, Honolulu (BPBM). Measurements and counts given here follow the methods outlined in (Randall and McCosker 1992). Proportional measurements are given as ratios of: standard length (SL; measured from the tip of the snout to the base of the caudal fin at the posterior edge of the hypural plate); head length (HL, measured from the median anterior point of the upper lip to the end of the longest opercular spine), or body depth (BD, measured as the maximum depth from the base of the spinous portion of the dorsal fin). Character values for the paratype are presented in parentheses, if different from those of the holotype. Meristics and measurements were compared with data obtained from the literature (Randall and McCosker 1992) for the six currently recognized species of *Luzonichthys*: *L. earlei (n= 26)*, *L. microlepis (n= 13)*, *L. taeniatus (n= 8)*, *L. waitei (n= 57)*, *L. whitleyi (n=33)*, and *L. williamsi (n= 8)*.

Tissue samples were obtained from the two individuals of *L. seaver*. Total genomic DNA was extracted from both samples using the 'HotSHOT' protocol (Meeker et al. 2007). A 690-bp fragment of the mtDNA cytochrome c oxidase 1 (CO1) region was amplified using modified primers from (Baldwin et al. 2009​): Fish-BCH (5'-ACTTCYGGGTGRCCRAARAATCA-3') and Fish-BCL (5'-TCAACYAATCAYAAAGATATYGGCAC-3'). Polymerase chain reaction (PCR) was performed in a 15 µl reaction containing 7.5 µl BioMix Red (Biolone Inc., Springfield, NJ, USA), 0.2 µM of each primer, 5-50 ng template DNA, and nanopure water (Thermo Scientific* Barnstead, Dubuque, IA, USA) to volume. PCR cycling parameters were as follows: initial 95°C denaturation for 10 min. followed by 35 cycles of 94°C for 30 sec, 55°C for 30 sec, and 72°C for 30 sec, followed by a final extension of 72°C for 10 min. PCR products were visualized using a 1.5% agarose gel with GelStarTM (Cambrex Bio Science Rockland, Inc., Rockland MA, USA) and then cleaned by incubating with 0.75 units of Exonuclease and 0.5 units of Shrimp Akaline Phosphate (ExoSAP; USB, Cleveland, OH, USA) per 7.5 µl of PCR product for 30 min. at 37°C followed by 85°C for 15 min. Sequencing was conducted in the forward and reverse direction using a genetic analyzer (ABI 3730XL, Applied Biosystems, Foster City, California) at the ASGPB Genomics Sequencing Facility at the University of Hawaii at Manoa. The sequences were aligned edited and trimmed to a common length using Geneious Pro v.6.1.6 DNA analysis software (Biomatters. ​http://www.geneious.com/​​). CO1 haplotypes were deposited in GenBank (accession numbers KP110513 and KP110514) and BOLD (dx.doi.org/10.5883/DS-LSE001).

## Taxon treatments

### Luzonichthys seaver

Copus, Ka'apu-Lyons, and Pyle 2015 sp. n.

urn:lsid:zoobank.org:act:68D04709-50C1-48D5-820C-FA4EC1BEF301

#### Materials

- **Type status:** Holotype. **Occurrence:** catalogNumber: 41205; recordedBy: Richard L. Pyle; individualID: afba0d7b-3eba-43a3-98a5-8edf341836d2; individualCount: 1; lifeStage: adult; preparations: 55% Isopropyl; disposition: in collection; **Taxon:** taxonID: 68d04709-50c1-48d5-820c-fa4ec1bef301; scientificNameID: 68d04709-50c1-48d5-820c-fa4ec1bef301; acceptedNameUsageID: 68d04709-50c1-48d5-820c-fa4ec1bef301; parentNameUsageID: 5b101671-671b-4200-8b57-17c8548a7180; originalNameUsageID: 68d04709-50c1-48d5-820c-fa4ec1bef301; nameAccordingToID: edb2b394-7d15-42a5-ac89-d979af29aaa7; namePublishedInID: edb2b394-7d15-42a5-ac89-d979af29aaa7; scientificName: *Luzonichthys seaver* Copus, Ka'apu-Lyons and Pyle; acceptedNameUsage: *Luzonichthys seaver* Copus, Ka'apu-Lyons and Pyle sec Copus, Ka'apu-Lyons and Pyle; parentNameUsage: *Luzonichthys* Herre 1936; originalNameUsage: *Luzonichthys seaver* Copus, Ka'apu-Lyons and Pyle; nameAccordingTo: Copus J, Ka'apu-Lyons C, Pyle R (2015) *Luzonichthys seaver*, a new species of Anthiinae (Perciformes, Serranidae) from Pohnpei, Micronesia. Biodiversity Data Journal 3: e4902.; namePublishedIn: Copus J, Ka'apu-Lyons C, Pyle R (2015) *Luzonichthys seaver*, a new species of Anthiinae (Perciformes, Serranidae) from Pohnpei, Micronesia. Biodiversity Data Journal 3: e4902.; higherClassification: Animalia, Deuterostomia, Chordata, Craniata, Gnathostomata, Actinopterygii, Perciformes, Percoidei, Serranidae, Anthiinae, *Luzonichthys*; kingdom: Animalia; phylum: Chordata; class: Actinopterygii; order: Perciformes; family: Serranidae; genus: Luzonichthys; specificEpithet: seaver; taxonRank: species; verbatimTaxonRank: Species; scientificNameAuthorship: Copus, Ka'apu-Lyons and Pyle; vernacularName: Seaver Splitfin; nomenclaturalCode: ICZN; **Location:** higherGeography: Pacific Ocean, Western Pacific Ocean, Micronesia, Caroline Islands, Senyavin (Pohnpei) Islands; waterBody: Pacific Ocean; islandGroup: Caroline Islands; island: Ahnd (Ant) Atoll; country: Federated States of Micronesia; countryCode: FM; stateProvince: Pohnpei; locality: southwest end; verbatimLocality: Pacific Ocean, Western Pacific Ocean, Micronesia, Caroline Islands, Senyavin (Pohnpei) Islands, southwest end of Ahnd (Ant) Atoll; verbatimDepth: 90-100m; minimumDepthInMeters: 90; maximumDepthInMeters: 100; decimalLatitude: 6.79018; decimalLongitude: 158.034245; geodeticDatum: WGS84; coordinateUncertaintyInMeters: 30; georeferenceProtocol: GPS; **Identification:** identifiedBy: Richard L. Pyle; dateIdentified: 08/01/2014; **Event:** samplingProtocol: Hand net; eventDate: 07/10/2014; year: 2014; month: 7; day: 10; habitat: rock outcrop along steep slope at top of drop-off; **Record Level:** modified: 2014-10-29T23:30:00Z; language: en; collectionID: http://biocol.org/urn:lsid:biocol.org:col:1001; institutionCode: BPBM; collectionCode: I; ownerInstitutionCode: BPBM; basisOfRecord: PreservedSpecimen
- **Type status:** Paratype. **Occurrence:** catalogNumber: 41206; recordedBy: Richard L. Pyle; individualID: ff70b774-16f8-4469-8229-b2e0a9b655fa; individualCount: 1; lifeStage: adult; preparations: 55% Isopropyl; disposition: in collection; **Taxon:** taxonID: 68d04709-50c1-48d5-820c-fa4ec1bef301; scientificNameID: 68d04709-50c1-48d5-820c-fa4ec1bef301; acceptedNameUsageID: 68d04709-50c1-48d5-820c-fa4ec1bef301; parentNameUsageID: 5b101671-671b-4200-8b57-17c8548a7180; originalNameUsageID: 68d04709-50c1-48d5-820c-fa4ec1bef301; nameAccordingToID: edb2b394-7d15-42a5-ac89-d979af29aaa7; namePublishedInID: edb2b394-7d15-42a5-ac89-d979af29aaa7; scientificName: *Luzonichthys seaver* Copus, Ka'apu-Lyons and Pyle; acceptedNameUsage: *Luzonichthys seaver* Copus, Ka'apu-Lyons and Pyle sec Copus, Ka'apu-Lyons and Pyle; parentNameUsage: *Luzonichthys* Herre 1936; originalNameUsage: *Luzonichthys seaver* Copus, Ka'apu-Lyons and Pyle; nameAccordingTo: Copus J, Ka'apu-Lyons C, Pyle R (2015) *Luzonichthys seaver*, a new species of Anthiinae (Perciformes, Serranidae) from Pohnpei, Micronesia. Biodiversity Data Journal 3: e4902.; namePublishedIn: Copus J, Ka'apu-Lyons C, Pyle R (2015) *Luzonichthys seaver*, a new species of Anthiinae (Perciformes, Serranidae) from Pohnpei, Micronesia. Biodiversity Data Journal 3: e4902.; higherClassification: Animalia, Deuterostomia, Chordata, Craniata, Gnathostomata, Actinopterygii, Perciformes, Percoidei, Serranidae, Anthiinae, *Luzonichthys*; kingdom: Animalia; phylum: Chordata; class: Actinopterygii; order: Perciformes; family: Serranidae; genus: Luzonichthys; specificEpithet: seaver; taxonRank: species; verbatimTaxonRank: Species; scientificNameAuthorship: Copus, Ka'apu-Lyons and Pyle; vernacularName: Seaver Splitfin; nomenclaturalCode: ICZN; **Location:** higherGeography: Pacific Ocean, Western Pacific Ocean, Micronesia, Caroline Islands, Senyavin (Pohnpei) Islands; waterBody: Pacific Ocean; islandGroup: Caroline Islands; island: Ahnd (Ant) Atoll; country: Federated States of Micronesia; countryCode: FM; stateProvince: Pohnpei; locality: southwest end; verbatimLocality: Pacific Ocean, Western Pacific Ocean, Micronesia, Caroline Islands, Senyavin (Pohnpei) Islands, southwest end of Ahnd (Ant) Atoll; verbatimDepth: 90-100m; minimumDepthInMeters: 90; maximumDepthInMeters: 100; decimalLatitude: 6.79018; decimalLongitude: 158.034245; geodeticDatum: WGS84; coordinateUncertaintyInMeters: 30; georeferenceProtocol: GPS; **Identification:** identifiedBy: Richard L. Pyle; dateIdentified: 08/01/2014; **Event:** samplingProtocol: Hand net; eventDate: 07/10/2014; year: 2014; month: 7; day: 10; habitat: rock outcrop along steep slope at top of drop-off; **Record Level:** modified: 2014-10-29T23:30:00Z; language: en; collectionID: http://biocol.org/urn:lsid:biocol.org:col:1001; institutionCode: BPBM; collectionCode: I; ownerInstitutionCode: BPBM; basisOfRecord: PreservedSpecimen

#### Description

Dorsal rays X,16, the first two soft rays simple; anal rays III,7, the first spine very small and difficult to detect; first ray simple; pectoral rays 21 (19), the upper most and lower most rays simple; branched pelvic rays I,5; branched caudal rays 13; simple upper and lower segmented caudal rays 4; upper and lower procurrent caudal rays 13; lateral line scales 63 (64); scales above lateral line to origin of dorsal fin 5; scales below lateral line to origin of anal fin 12; gill rakers 8+19 (8 +18) (Table 1).

Body moderately elongate, the depth 3.86 (4.6) in SL, and compressed, the width 1.83 (1.25) in BD; head length 3.54 (3.41) in SL; snout short and bluntly rounded, 4.0 (3.86) in HL. Orbit diameter 3.43 (3.8) in HL; the least width of interorbital space 3.0 (3.38) in HL; caudal peduncle depth 2.4 (2.45) in HL; caudal peduncle length about twice its depth, 1.09 (1.23) in HL. Mouth terminal and oblique, the maxilla reaching posterior to rear edge of pupil but not posterior to rear edge of orbit; the upper jaw length 1.6 (1.93) in HL; corners of maxilla rounded, its greatest depth about equal to pupil diameter. Opercle with 2 flat spines, the lower acute, in line with center of eye and opercular flap, the upper spine at dorsal end of gill opening poorly developed. Lateral line only slightly arched above pectoral fin, gradually descending below soft portion of dorsal fin, straightening toward the peduncular region; scales on body ctenoid; head scaled except snout; dorsal, anal, and pelvic fins naked; caudal fin with small scales extending about three-fourths distance to posterior margin; basal fifth of pectorals with small scales. Origin of dorsal fin above eighth lateral-line scale; first dorsal spine short, 8.0 (9.0) in head; fourth dorsal spine longest, 2.4 (2.45) in HL; longest dorsal soft ray 2.4 (2.45) in HL; origin of anal fin below base of sixth dorsal soft ray; first anal spine very short, about 12(13.5) in HL; second anal spine 6.0 (6.75) in HL; first anal soft ray very slender and only partly segmented, 2.4 (2.7) in HL; longest anal soft ray 2.0 (2.45) in HL; caudal fin forked, with filamentous rays, the fin length 4.05 (3.83) in SL, the caudal concavity 8.5 (8.36) in SL; middle pectoral rays longest, 3.86 (4.38) in SL; origin of pelvic fins below lower base of pectorals; second pelvic soft ray longest, 5.67 (6.57) in SL (Table 2).

**Figure 1.**
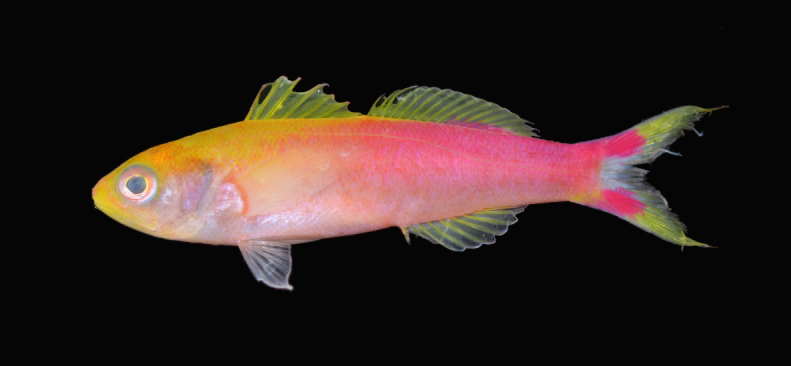
Holotype of *Luzonichthys seaver*, **BPBM 41205**, Pohnpei, Micronesia. Photo: Brian D. Greene.

Color of holotype in life: head excluding operculum predominately yellow (many scales with yellow margins and pink centers), yellow extending posteriorly on upper half of body from a line starting at approximately the tip of the opercular flap and top of pectoral fins to the eighth dorsal ray, fading to bright pink posteriorly. Operculum to lower half of body salmon pink. Pectoral and pelvic fins pale. Dorsal fins yellow with bases of rear dorsal rays pink. Anal fin rays yellow with pale membranes. Upper and lower base of caudal fin pink, extending posteriorly to approximately halfway to the tips; center of base of caudal fin white, fading to pale; posterior half of caudal fin yellow. Lower base of caudal peduncle with yellow band (Fig. 1).

Color of holotype in alcohol: Pale, all fins colorless except the caudal which has purple spots on the base of each of the upper and lower segments, the upper extending anteriorly, dorsally on the caudal peduncle.

#### Diagnosis

Dorsal rays X,16; anal rays III,7; pectoral rays 19-21; lateral line scales 63-54; gill rakers 8+18-19; Body moderately elongate, the depth 3.86-4.6 in SL; head length 3.41-3.54 in SL; snout 3.86-4.0 in HL; caudal fin forked, with filamentous rays, caudal concavity 8.36-8.5 in SL; pectoral fins 3.86-4.38 in SL; pelvic fins 5.67-6.54 in SL.

#### Etymology

Named *seaver*, as a noun in apposition, for the Seaver family in recognition of support from the Seaver Institute for marine research.

#### Distribution

Type specimens of *L. seaver* were collected from Pohnpei, Micronesia. A single larva of what may be this species (98.2-99.5% similarity at CO1) was collected in Moorea, French Polynesia (BOLD: FPFLB281-12; GenBank: KJ967845.1; Hubert et al. 2014​), but the taxonomic identity of the Moorea specimen could not be confirmed by the authors. Therefore, it is possible that adults of this species will be discovered in Moorea and other locations across the Pacific.

#### Taxon discussion

**Figure 2.**
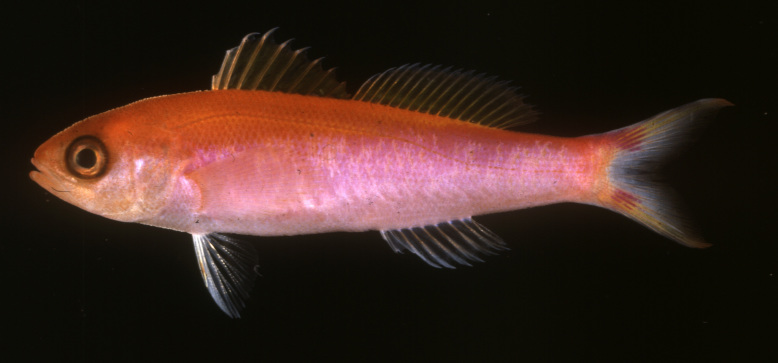
Luzonichthys earlei, from the Hawaiian Islands. Photo: John E. Randall.

*Luzonichthys seaver* is distinct from all other species within the genus in life coloration. It is most similar in color to *L. earlei* (Fig. 2); however, it differs from that species in coloration of head, upper half of body, and dorsal and anal fins (yellow in *L. seaver*, compared with orange in *L. earlei*), and in the distinctive color pattern on the caudal fin (bright pink blotches on the base of both caudal lobes with bright yellow distally in *L. seaver*, compared with drab, diffuse orange blotches and pale yellow distal caudal lobes in *L. earlei*). We have examined enough individuals of *L. earlei* from many localities to confirm that these color characteristics do not vary signigicantly within that species. Morphologically, it differs from all other species except *L. earlei* in number of lateral line scales (63-64, compared with 51-60 or 65-78 for other species), and from all other species except *L. whitleyi* in caudal concavity (8.4-8.5 in HL, compared with 4.0-8.3 for other species). It further differs from all other species except *L. earlei*, *L. waitei* and *L. taeniatus* in number of gill rakers (8+18-19, compared with 7-10+20-23 for other species), and from *L. williamsi* and *L. microlepis* in number of anal-fin spines and rays (III, 7 compared with II, 9), as well as snout length, orbit diameter, and caudal peduncle depth (Table 3). *Luzonichthys seaver* is further distinct from *L. williamsi* in its body depth and pectoral fin length, and from *L. taeniatus* in snout length, caudal peduncle depth, and longest dorsal spine (Table 3). Overall, *L. seaver* is most similar morphologically to *L. earlei* and *L. whitleyi*; however, in addition to character differences outlined above, it can be further distinguished from these two species by caudal peduncle depth (both species) snout length (*L. whitleyi*), and head length (*L. earlei*) (Table 3​). Molecular data are not used for comparative purposes in this description because these data do not exist for any of the other species of *Luzonichthys*. The CO1 barcodes produced in this study represent the first sequences publicly available for this genus, aside from the afore mentioned and previously unclassified larval specimen from Moorea.

## Supplementary Material

XML Treatment for Luzonichthys seaver

## Figures and Tables

**Table 1.** Counts of dorsal rays, anal rays, pectoral rays, lateral line scales, and gill rakers of the species of *Luzonichthys*.

	Dorsal rays	Anal rays	Pectoral rays	Lateral line scales	Gill rakers
*L. seaver*	X,16	III,7	19-21	63-64	8+18-19
*L. earlei*	X,16-17	III,7	19-21	59-68	6-9+19-22
*L. microlepis*	X,16	II,9	21-22	70-76	7-8+21-23
*L. taeniatus*	X,16	III,7	19	56-60	7+19
*L. waitei*	X,15-17	III,7	17-21	51-59	7-10+19-22
*L. whitleyi*	X,16	III,7	19-22	65-74	7-9+20-23
*L. williamsi*	X,16	II,9	21-23	70-78	7-8+21-22

**Table 2.** Proportional measurements of type specimens of *Luzonichthys seaver* expressed as percentages of standard length

	**Holotype BPBM 41205**	**Paratype BPBM 41206**
Standard length (mm)	42.5	46
Body depth	25.9	21.7
Body width	14.1	17.4
Head length	28.2	29.3
Snout length	7.1	7.6
Orbit diameter	8.2	8.7
Interorbital width	9.4	8.7
Upper jaw length	17.6	15.2
Caudal peduncle depth	11.8	12.0
Caudal peduncle length	25.9	23.9
Predorsal length	35.3	34.8
Preanal length	62.4	70.0
Prepelvic length	33.0	32.6
First dorsal spine	3.5	3.3
Second dorsal spine	9.4	8.7
Third dorsal spine	10.6	8.7
Forth dorsal spine	11.8	12.0
Longest dorsal spine	11.8	12.0
First anal spine	2.4	2.2
Second anal spine	4.7	4.3
First anal ray	11.8	10.9
Longest anal ray	14.1	12.0
Caudal fin length	24.7	26.1
Caudal concavity	11.8	12.0
Pectoral fin length	25.9	22.8
Pelvic spine length	9.4	7.6
Pelvic fin length	17.6	15.2

**Table 3.** Comparison of selected morphological characters for species of *Luzonichthys*.

	**Character**	***L. seaver***	***L. earlei***	***L. microlepis***	***L. taeniatus***	***L. waitei***	***L. whitleyi***	***L. williamsi***
**Body depth**	in SL	3.9-4.6	3.6-4.1	4.1-4.4	3.7-4.0	3.3-3.8	4.15-5.0	5.2-5.4
**Head length**	in SL	3.4-3.5	3.15-3.4	3.5-3.7	3.3-3.5	3.0-3.6	3.4-3.8	3.4-3.5
**Snout length**	in HL	3.9-4.0	3.9-4.3	4.0-4.3	4.2-4.4	3.7-4.1	4.2-4.8	4.3-4.6
**Orbit diameter**	in HL	3.4	3.1-3.9	4.1-4.4	3.0-3.5	3.5-3.9	3.2-3.6	2.7-3.3
**Caudal peduncle depth**	in HL	2.4-2.5	2.6-2.9	2.5-2.7	1.5	2.3-2.6	2.5-2.9	2.8-2.9
**Caudal concavity**	in SL	8.4-8.5	6.1-7.3	6.0-6.8	5.1-5.5	4.0-5.6	5.2-9.0	6.7-8.3
**Longest dorsal spine**	in HL	2.4-2.5	2.1-2.6	2.4-2.8	2.2-2.4	2.2-2.6	2.0-2.6	2.2-2.5
**Pectoral fin length**	in SL	3.9-4.4	3.7-4.0	4.1-4.7	3.6-3.7	3.2-3.5	3.7-4.4	4.4-4.7
**Pelvic fin length**	in SL	5.7-6.6	4.4-5.2	5.0-5.8	4.6-4.9	3.9-4.7	4.6-6.0	4.8-5.8
